# Supplementary material for: mRNA nuclear retention reduces AMPAR expression and promotes autistic behavior in UBE3A-overexpressing mice
Source: EMBO Rep. 2024 Feb 5;25(3):21. doi: 10.1038/s44319-024-00073-1 (PMC10933332; doi:10.1038/s44319-024-00073-1)
Supplement: Supplementary file 1 — Appendix [file 44319_2024_73_MOESM1_ESM.pdf]

mRNA nuclear retention leads to reduced AMPA receptor expression and neuronal activity in UBE3A-dependent ASD

Yuan Tian[1], Feiyuan Yu[1], Eunice Yun[1], Jen-Wei Lin[1], Heng-Ye Man[1, 2, 3] *

[1]Department of Biology, Boston University, 5 Cummington Mall, Boston, MA 02215, USA

[2]Department of Pharmacology, Physiology & Biophysics, Boston University School of Medicine, 72 East Concord St., Boston, MA 02118, USA

[3]Center for Systems Neuroscience, Boston University, 610 Commonwealth Ave, Boston, MA 02215, USA

*Correspondence should be addressed to: hman@bu.edu

Appendix Figure S1…………………………..…………………………..…………Page 2

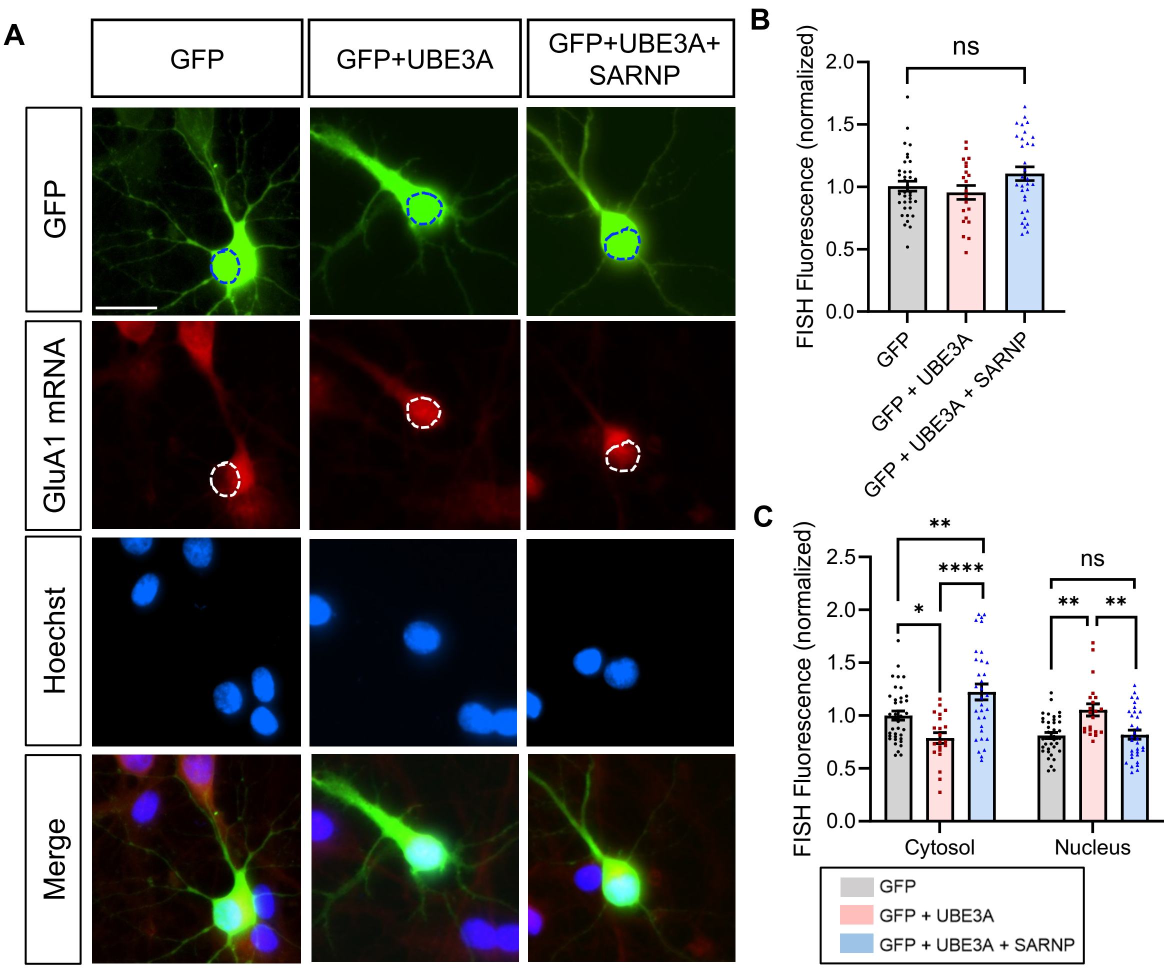

Appendix Figure S1. UBE3A overexpression induces GluA1 mRNA nuclear retention in primary rat neurons, which can be rescued by SARNP.

(A) GluA1 RNA FISH (red) was performed in rat primary cortical neurons transfected with GFP, GFP+UBE3A or GFP+UBE3A+SARNP. Scale bars, 25 µm.

(B) UBE3A overexpression didn't affect the total level of GluA1 mRNA in the soma.

(C) UBE3A transfection resulted in a reduction of cytosolic GluA1 mRNA and an increase in nuclear GluA1 mRNA. Both of these changes were reversed by SARNP co-transfection.

GFP: n=37, GFP+UBE3A: n=21, GFP+UBE3A+SARNP: n=32. Mean ± SEM. *$p<0.05$; **$p<0.01$; ****$p<0.0001$; ns = not significant. In (B) One-way ANOVA with Bonferroni's multiple comparisons test. In (C) Two-way ANOVA with Bonferroni's multiple comparisons test.
