# Supplementary material for: mRNA nuclear retention reduces AMPAR expression and promotes autistic behavior in UBE3A-overexpressing mice
Source: EMBO Rep. 2024 Feb 5;25(3):21. doi: 10.1038/s44319-024-00073-1 (PMC10933332; doi:10.1038/s44319-024-00073-1)
Supplement: Supplementary file 3 — Expanded View Figures [file 44319_2024_73_MOESM3_ESM.pdf]

Expanded View Figures

Figure EV1. Validation of GluA1 mRNA FISH.

(**A**) GluA1 RNA FISH (red) in DIV14 primary rat neurons transfected with either SCR-GFP, shGluA1-GFP to knock down GluA1, or GluA1-GFP to overexpress GluA1. Hoechst stained nucleus. Arrows point to the transfected cells. Scale bars, 25 μm. (**B**) Quantification showed a reduction in the FISH fluorescent intensity of GluA1 mRNA following GluA1 knock down by shRNA; and increased FISH fluorescent intensity of GluA1 mRNA following GluA1 overexpressing. (**C**) Knocking down or overexpressing GluA1 mainly affected GluA1 mRNA level in the cytosol rather than that in the nucleus. Data information: SCR-GFP: $n = 35$, shGluA1-GFP: $n = 36$, GluA1-GFP: $n = 33$. Mean ± SEM. $^{**}p < 0.01$; $^{***}p < 0.001$; $^{****}p < 0.0001$; ns = not significant. In (**B**), one-way ANOVA with Bonferroni's multiple comparisons test. In (**C**), two-way ANOVA with Bonferroni's multiple comparisons test.

© The Author(s)

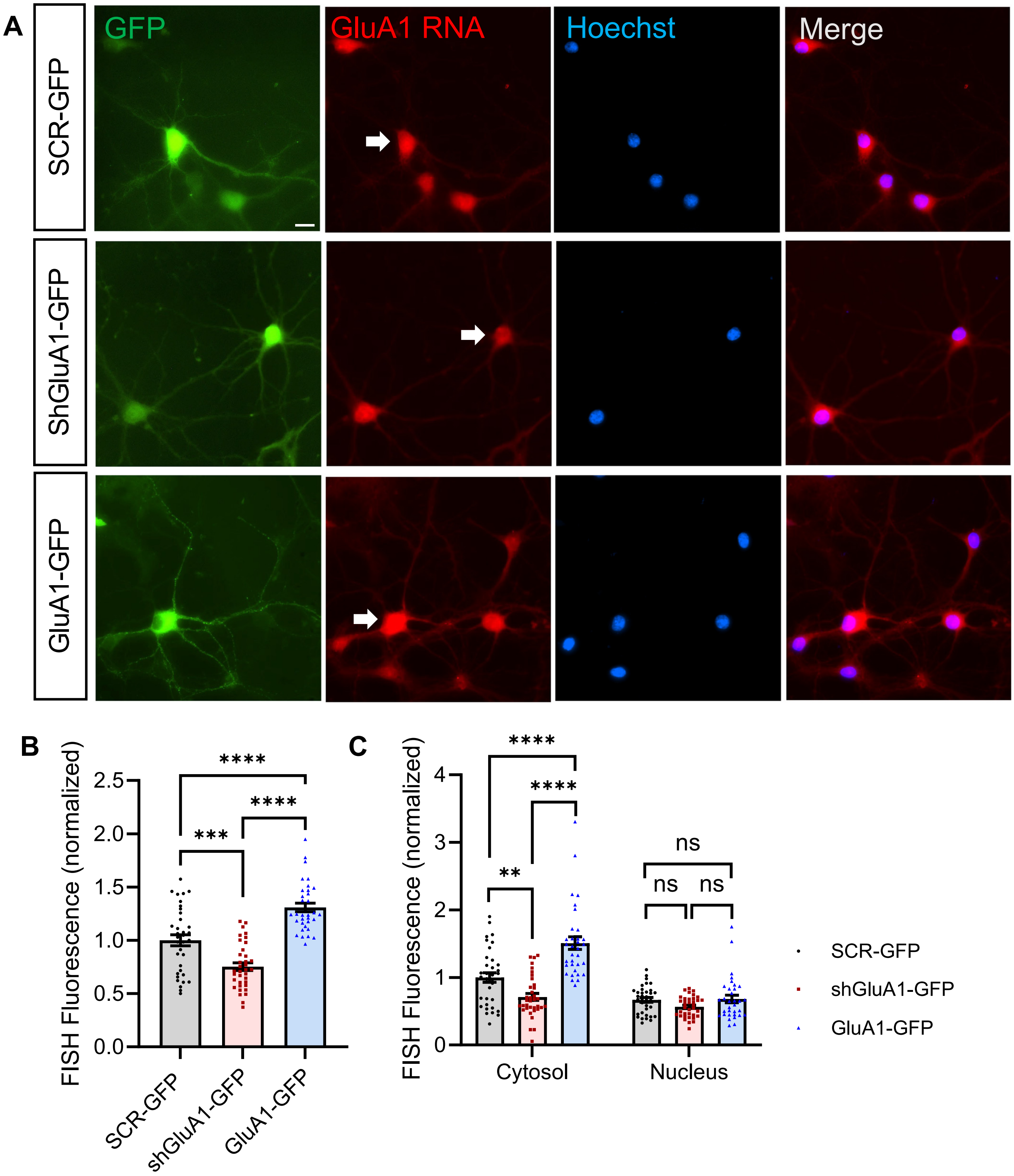
A
SCR-GFP
ShGluA1-GFP
GluA1-GFP
GFP
GluA1 RNA
Hoechst
Merge
B
FISH Fluorescence (normalized)
2.5
2.0
1.5
1.0
0.5
0.0
****
****
***
SCR-GFP
shGluA1-GFP
GluA1-GFP
C
FISH Fluorescence (normalized)
4
3
2
1
0
****
****
**
ns
ns
ns
Cytosol
Nucleus
SCR-GFP
shGluA1-GFP
GluA1-GFP

 © The Author(s)

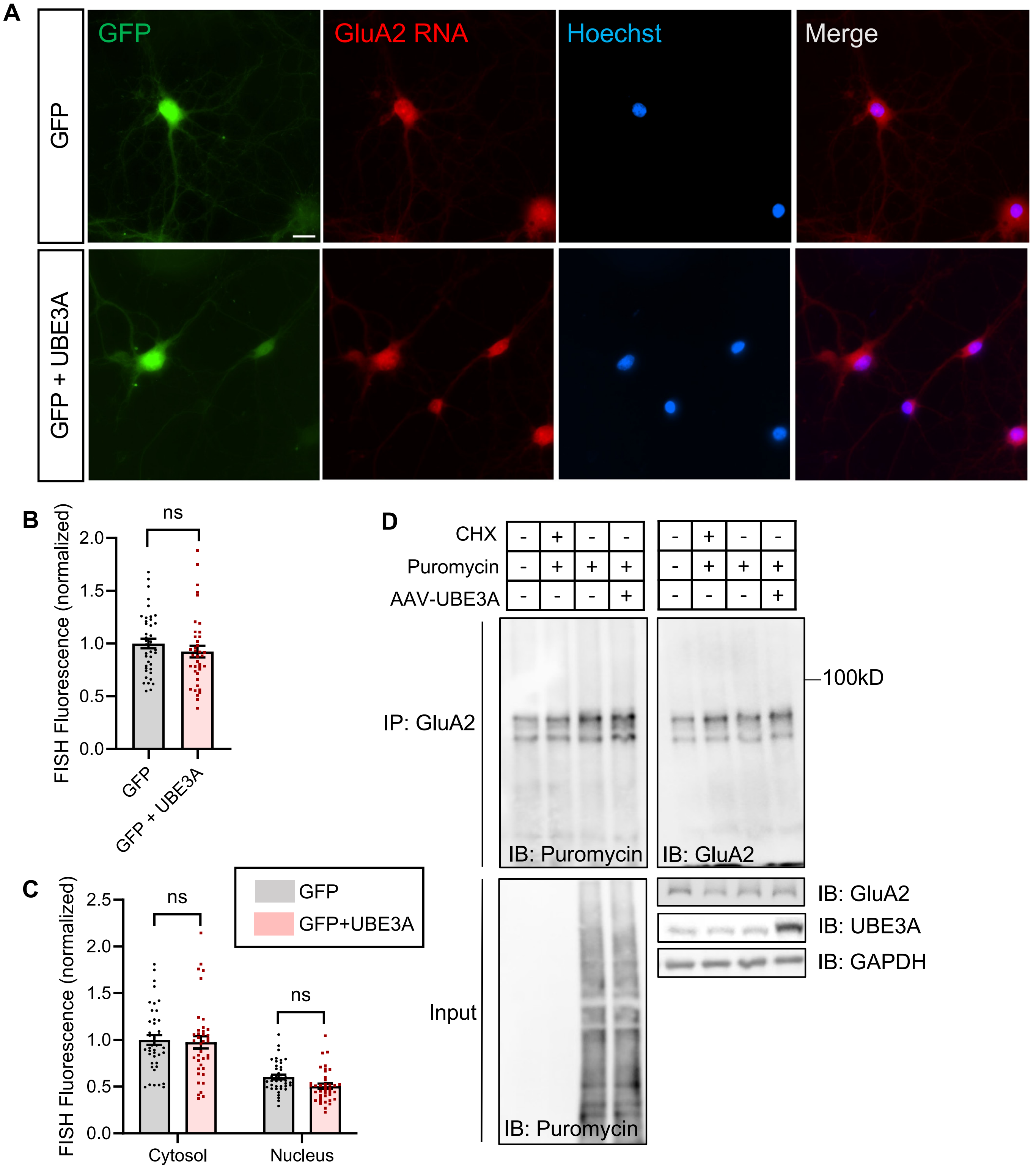
A
GFP
GluA2 RNA
Hoechst
Merge
GFP
GFP + UBE3A
B
ns
FISH Fluorescence (normalized)
GFP
GFP + UBE3A
C
ns
GFP
GFP+UBE3A
FISH Fluorescence (normalized)
Cytosol
Nucleus
D
CHX
Puromycin
AAV-UBE3A
IP: GluA2
100kD
IB: Puromycin
IB: GluA2
Input
IB: GluA2
IB: UBE3A
IB: GAPDH
IB: Puromycin

Figure EV2. GluA2 mRNA localization and GluA2 protein synthesis are intact with UBE3A overexpression.

(**A**) Primary rat neurons were transfected with either GFP or GFP + UBE3A, and GluA2 mRNA (red) was examined by RNA FISH at DIV14. The nucleus was stained with Hoechst. Scale bars, 25 μm. (**B**, **C**) Quantification of the GluA2 mRNA intensity showed that UBE3A overexpression did not alter the GluA2 mRNA levels in the soma, nor did it alter GluA2 mRNA distribution in the cytosol vs. nucleus. GFP: $n = 41$, GFP + UBE3A: $n = 39$. (**D**) Puromycin assay was performed to examine GluA2 protein synthesis in primary cortical neurons infected with GFP or UBE3A virus. The neurons were treated with DMSO or puromycin for 12 h followed by GluA2 immunoprecipitation and probing for puromycin. Cell lysates (input) were also probed to detect total protein levels. Data information: Mean ± SEM. ns = not significant. In (**B**), unpaired two-tailed *t* test. In (**C**), two-way ANOVA with Bonferroni's multiple comparisons test.

© The Author(s)

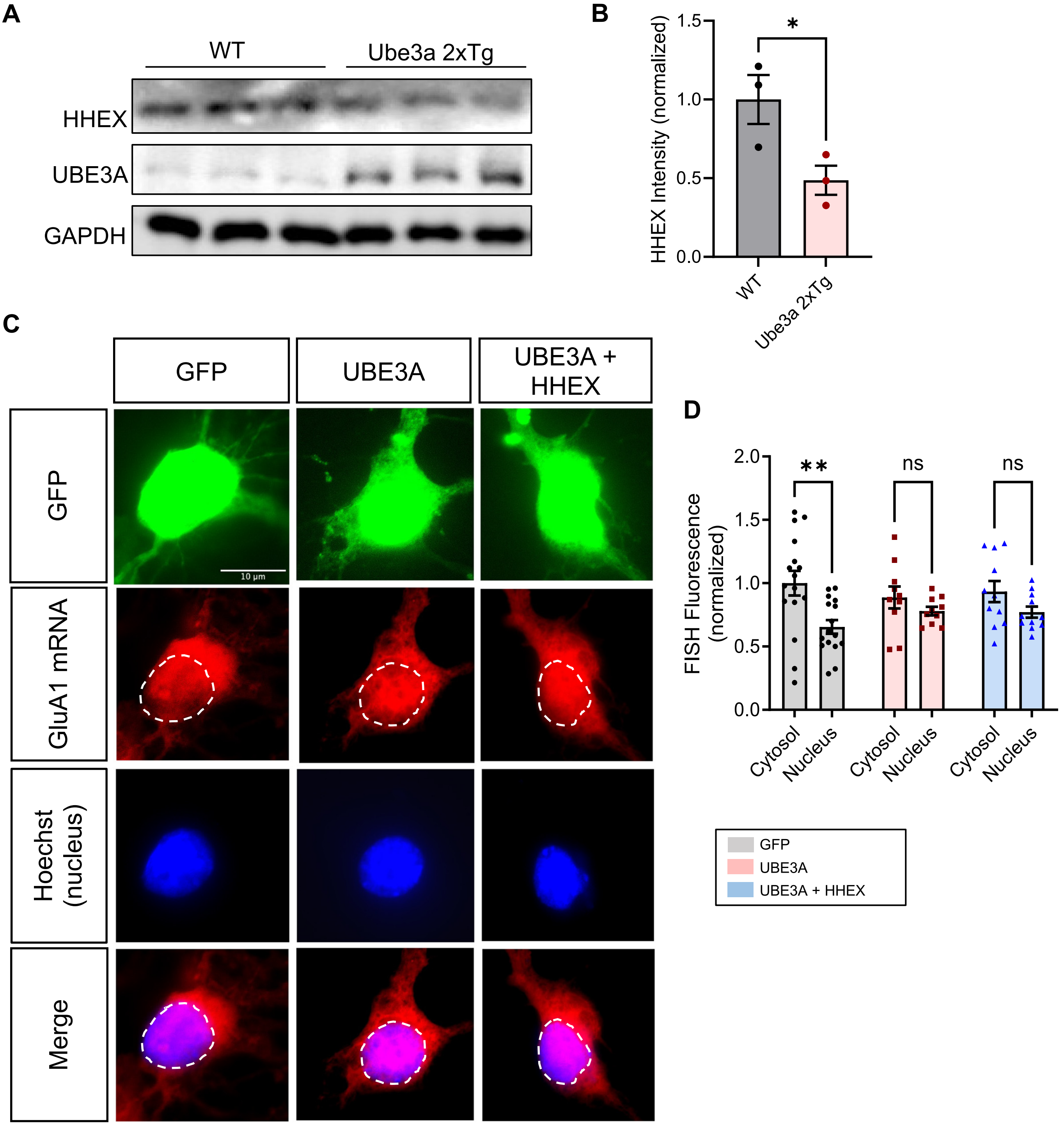

Figure EV3. Nuclear retention of GluA1 mRNA is not attributed to HHEX reduction in UBE3A-overexpression conditions.

(**A**) Western blot analysis revealed HHEX protein expression levels in brain lysates from WT and *Ube3a* 2xTg mice. (**B**) A notable decrease in HHEX was observed in *Ube3a* 2xTg mouse brains. $n = 3$ animals/group. (**C**) GluA1 RNA FISH (red) was conducted on rat primary cortical neurons that were transfected with either GFP, GFP + UBE3A, or GFP + UBE3A + HHEX. Scale bars, 10 μm. (**D**) UBE3A transfection resulted in nuclear retention of GluA1 mRNA, which was not rescued by HHEX co-transfection. GFP: $n = 16$, GFP + UBE3A: $n = 10$, GFP + UBE3A + HHEX: $n = 11$. Data information: Mean ± SEM. $^*p < 0.05$; $^{**}p < 0.01$; ns = not significant. In (**B**), unpaired two-tailed t test. In (**D**), two-way ANOVA with Bonferroni's multiple comparisons test.

© The Author(s)

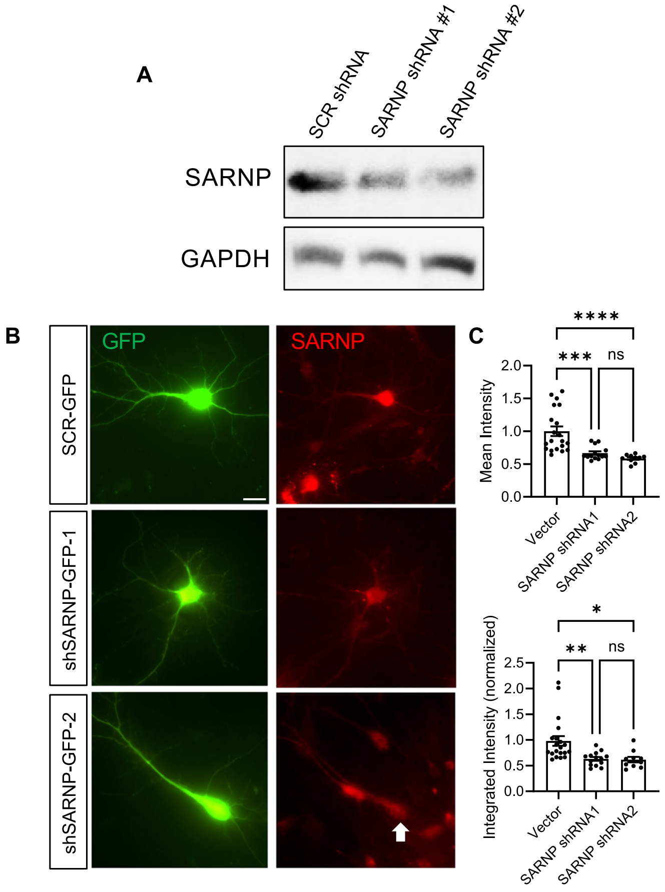

Figure EV4. SARNP shRNAs effectively reduce SARNP expression in HEK cells and neurons.

(**A**) Validation of SARNP shRNAs in HEK cells. HEK cells were transfected with SCR shRNA, SARNP shRNA #1, or SARNP shRNA #2. Both SARNP shRNA constructs reduced SARNP expression. (**B**) Validation of SARNP shRNAs in primary rat neurons. Neurons were transfected with SCR vector, SARNP shRNA #1, or SARNP shRNA #2. Arrows point to the transfected cells. Scale bars, 25 μm. (**C**) Mean intensity (top) and integrated intensity (bottom) of SARNP in the soma were significantly reduced by SARNP shRNA #1 and SARNP shRNA #2. SCR vector: $n = 20$, SARNP shRNA #1: $n = 15$, SARNP shRNA #2: $n = 10$. Data information: Mean ± SEM. *$p < 0.05$; **$p < 0.01$; ***$p < 0.001$; ****$p < 0.0001$; ns = not significant. One-way ANOVA with Bonferroni's multiple comparisons test.

 © The Author(s)

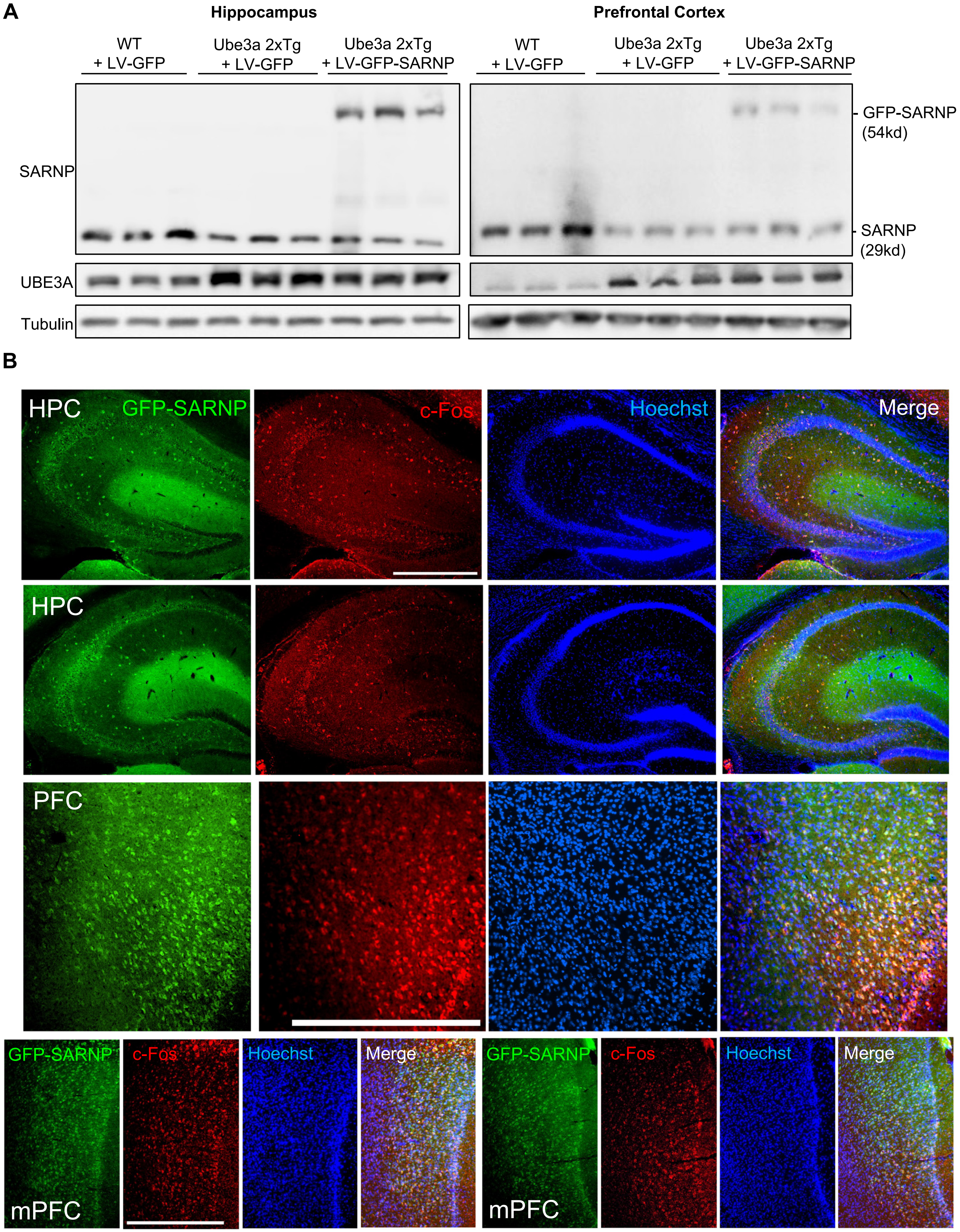
A
Hippocampus
Prefrontal Cortex
WT
+ LV-GFP
Ube3a 2xTg
+ LV-GFP
Ube3a 2xTg
+ LV-GFP-SARNP
SARNP
UBE3A
Tubulin
GFP-SARNP
(54kd)
SARNP
(29kd)
B
HPC
GFP-SARNP
c-Fos
Hoechst
Merge
PFC
mPFC

Figure EV5. LV-GFP-SARNP virus expression in *Ube3a* 2xTg mouse brain.

(**A**) In P40 *Ube3a* 2xTg mice injected with LV-GFP-SARNP virus, lysates from the hippocampus and prefrontal cortex demonstrated robust GFP-SARNP expression. (**B**) Using immunohistochemistry, colocalization of GFP-SARNP expression with c-Fos was observed in the hippocampus and prefrontal cortex of P40 *Ube3a* 2xTg mice injected with LV-GFP-SARNP. Scale bars, 500 μm.

 © The Author(s)
